# Multiplexed paper-based assay for personalized antimicrobial susceptibility profiling of Carbapenem-resistant *Enterobacterales* performed in a rechargeable coffee mug

**DOI:** 10.1038/s41598-022-16275-3

**Published:** 2022-07-14

**Authors:** Taylor Oeschger, Lauren Kret, David Erickson

**Affiliations:** 1 Meinig School of Biomedical Engineering, Cornell University, Ithaca, NY 14853 USA; 2 Sibley School of Mechanical and Aerospace Engineering, Cornell University, Ithaca, NY 14853 USA; 3 Division of Nutritional Science, Cornell University, Ithaca, NY 14853 USA

**Keywords:** Diagnosis, Antimicrobial resistance, Biomedical engineering

## Abstract

The increasing prevalence of antibiotic resistance threatens to make currently treatable bacterial diseases deadly again. As drug resistance rises, antibiotic susceptibility testing needs to adapt to allow for widespread, individualized testing. Paper-based diagnostics offer low-cost, disposable alternatives to traditional time consuming and costly in-house methods. Here, we describe a paper-based microfluidic device, called the Bac-PAC, capable of categorizing the antibiotic susceptibly of individual strains of Carbapenem-resistant *Enterobacterales*. Each chip provides a colored readout with actionable susceptibility classification of three antibiotics, thus maximizing the chances of identifying a viable therapy. We verified the technology on thirty bacterial strains with two dyes using six clinically relevant antibiotics. We demonstrated that the dried tests are stable for one month and can be incubated in a rechargeable coffee mug that reduces the need for external infrastructure.

## Introduction

Antimicrobial resistance (AMR) is immediate pressing global threat. In 2016, AMR caused 700,000 deaths worldwide, and models predict an increase of up to 10 million deaths per year by 2050, resulting in 100 trillion USD lost in global production^1^. Carbapenem-resistant *Enterobacterales* (CRE) is a highly antibiotic resistant group of organisms dubbed the “nightmare bacteria” because of their estimated annual deaths, prevalence in hospitals, and rapid transfer of antibiotic resistance genes^2^. Many experts in AMR broadly recommended that antimicrobial susceptibility testing (AST) be performed on an individual basis so that personalized treatments can be prescribed^3,4^. This would prevent the misuse of last-line antibiotics and delaying the onset of full resistance.

The gold standard method of AST is broth serial dilution, where solutions of antibiotics diluted two-fold are prepared, spiked with bacteria, and the minimum inhibitory concentration is obtained^5^ and classified based on breakpoints as determined by the Clinical and Laboratory Standards Institute (CLSI)^6^. While this produces reliable, reproducible, quantitative results, this method is also limited by its tedious preparation, high cost, and laboratory requirments^5^. It is currently impractical to preform broth serial dilutions for multiple antibiotics for several patients without the aid of automated equipment. Other methods for AST may include automated devices, premade 96-well plates, E-test gradient strip diffusion, or agar disk diffusion. While these technologies are logistically simpler than broth dilution, they may be more expensive, bulkier, or require refrigerated agar plates making them unamenable to low resource settings. When none of these methods are available, clinicians may prescribe patients an antibiotic treatment based on infection source or current regional resistance trends^7,8^.

The consensus Review on Antimicrobial Resistance concluded that “the solution to the problem (of AMR) must work for the world and benefit as many people as possible, not one country or one group of countries,” and thus “the solutions should be cost-effective, affordable and support economic development”^1^. This is especially important for low- and middle-income countries that lack the resources or infrastructure necessary to perform drug susceptibility testing^9,10^. It is therefore necessary to develop a low-cost point-of-care diagnostic to accurately predict effective antibiotics for treating a patient based on their individual infection resistance profile. Point-of-care diagnostic devices should be affordable, portable, robust, designed to work in a wide variety of settings, use small sample volumes, and have a quick turnaround time^11–13^. Paper microfluidics specifically offer several additional benefits: they are disposable, require no power supply, and are easy to scale for mass manufacturing^11,14,15^. Such a test could provide a global picture of emerging AMR, guide public health and policy, and improve individual patient outcomes.

Here we introduce the Bac-PAC: **Bac**terial **P**aper **A**ntibiotic Susceptibly Testing **C**hip. The Bac-PAC is a paper microfluidic chip capable of giving colored readouts visible to the naked eye corresponding to the susceptibly of a patient-specific bacterial strain. This work improves on our previous publication by Wang and Erickson^16^ through (1) multiplexing to include three antibiotics while maintaining semi-quantitative readouts, (2) exploration of additional colorimetric dyes that demonstrated better performance with some antibiotics, (3) proof of concept validation with 30 bacterial strains from 12 different species contained in the “*Enterobacterales* Carbapenem Breakpoint" panel^17^ (Supp. Figs. 1, 2), (4) implementation of packaging to store the device at different temperatures for extended periods of time, and (5) the incorporation of a low-cost rechargeable SmartMug in place of a commercial incubator. Altogether, the Bac-PAC assay described herein demonstrates the first multiplexed, colored AMR assay on paper that is optimized for clinically actionable information in low resource settings.

## Results

### Development of the Bac-PAC

The Bac-PAC is a wax printed paper-microfluidic, based on the original design described by Wang and Erickson^16^, where bacteria are placed in the center and diffuse radially into ten wells containing three antibiotics of interest at three concentrations each, plus one positive control with no antibiotics. Antibiotic concentrations increase in a counterclockwise direction. After incubation, the wells change color to indicate bacterial replication and a failure of the antibiotic to inhibit the infection. Zero or one wells changing indicates a susceptible sample, while two wells and three wells changing implies an intermediate and resistant sample, respectively. Simply put, less color change indicates more susceptible bacteria.

**Figure 1:**
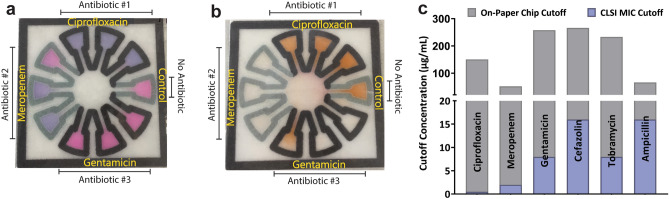
Bac-PAC design. (**a**) Example test using PrestoBlue dye displaying ciprofloxacin susceptible, meropenem susceptible, and gentamicin resistant results. (**b**) Example test using XTT dye displaying ciprofloxacin resistant, meropenem susceptible, and gentamicin susceptible results. (**c**) Concentrations used on paper chip (top, grey) compared to CLSI MIC liquid culture cut-offs (bottom, blue).

Prior to incubation, water is added to each of the four corners and the entire chip is sealed in a transparent film. This increases humidity, encourages bacterial growth, prevents contamination, and contains all biohazards. The multiplexed design includes three antibiotics on a single chip, thus producing more clinically actionable information from a single test compared to the previous system^16^. We compared resazurin-based PrestoBlue dye (Fig. 1a) with the tetrazolium dye XTT (Fig. 1b). PrestoBlue is a redox dye that starts as blue colored resazurin and is reduced to resorufin, a pink product, or further reduced to dihydroresorufin, a colorless product^18,19^. In contrast, XTT in solution starts colorless and is reduced to a vibrant orange, especially in the presence of additional electron acceptors such as phenazine methosulfate.

Antibiotics were selected using the CLSI M100 Performance Standards for Antimicrobial Susceptibility testing^6^. All four Group A antibiotic agents considered to be appropriate for inclusion in routine, primary testing panel were used. These included ampicillin, cefazolin, gentamicin, and tobramycin. Additionally, we chose two Group B antibiotics which are recommended to be included in testing if failure to agents in Group A is observed. The Group B agents selected were meropenem and ciprofloxacin due to their unique antibiotic classes compared to those in Group A.

We found that the concentration of antibiotics used on the Bac-PAC needed to be much higher than that used in traditional liquid MIC testing. Therefore, the concentration of each antibiotic on paper corresponding to susceptible, intermediate, and resistant cutoffs had to be identified experimentally. For each antibiotic, at least one strain from the panel was selected that contained susceptible, intermediate, and resistant gold standards and the antibiotic concentration were adjusted, or “tuned”, on paper until the categorization aligned. Overall, the intermediate cut-off level varied between 50 and 250 µg/mL, an average of 75-fold higher than the CLSI MIC breakpoints of 0.5 to 16 µg/mL^6^ (Fig. 1c). Gentamicin and cefazolin had an intermediate cut-off of 250 µg/mL, tobramycin 225 µg/mL, ciprofloxacin 150 µg/mL, and ampicillin and meropenem 50 µg/mL.

This magnitude difference in antibiotic concentrations warrants further investigation. Agar disk used for disk diffusion antibiotic susceptibility testing are loaded with high concentrations of antibiotics because the antibiotics must diffuse into the agar before contacting the bacteria. However, here the antibiotics should not be diffusing, and contact occurs at the site where antibiotics were dried. We suspect that higher concentrations of antibiotics are needed because of the higher bacterial inoculums needed to obtain growth and colored readouts on the paper format. Additionally, some antibiotics may be leaking to the center of the chip. The effectiveness of the antibiotics could also be decreased by the drying process or interaction with the chromatography paper itself. Despite possible antibiotic diffusion across the chip, antibiotics are not contaminating adjacent wells as can be seen from positive controls which are located directly besides the highest concentration of one of the antibiotics.

For analysis of results, discrepancies were separated into minor (a false resistant or susceptible result for an intermediate isolate or a false intermediate result), major (false resistant), and very major (false susceptible) in accordance with CLSI^6^ and the FDA^20^ guidelines. False resistant major discrepancies could cause a viable treatment option to be missed; however, this is preferable to a very major false susceptible in which the wrong treatment might be prescribed. The overall aim was to reduce all discrepancies, with an emphasis on very major discrepancies.

### Four-fold dilution is more ideal than two- or six- fold

**Figure 2:**
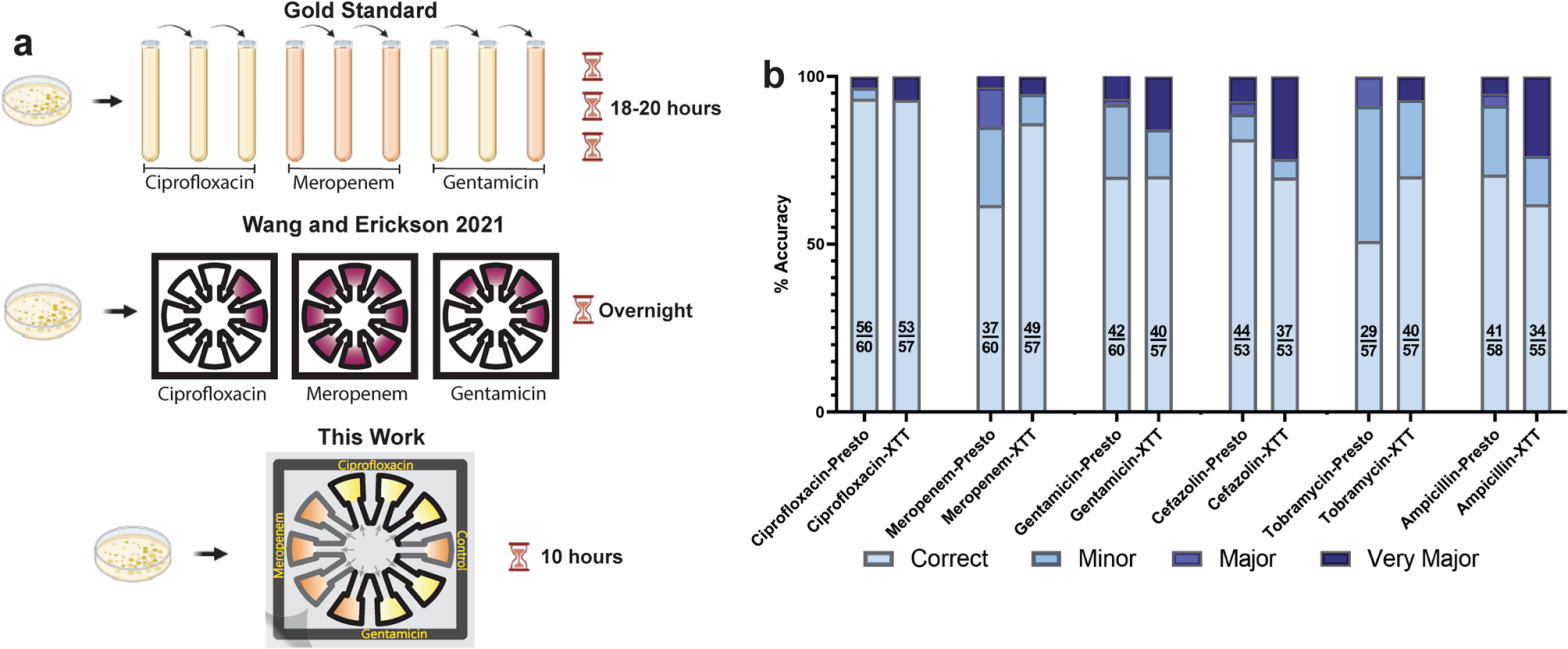
Bac-PAC compared to other technologies (**a**) Comparison of time and equipment of broth dilution (top), previous work (middle) and this work (bottom) (**b**) Bac-PAC four-fold dilutions results. Fractions represent number of correct samples over number of total samples.

In designing the Bac-PAC, we sought to maximize categorical agreement and to minimize major and very major discrepancies while also spanning the widest range of antibiotic concentrations possible. Gold standard broth microdilution testing involves adding bacteria to broth containing antibiotics that are diluted two-fold until growth of the bacteria ceases (Fig. 2a). These tests utilize eight to ten different antibiotic concentrations in a single test; however, for the Bac-PAC this was limited to three concentrations to minimize the device footprint. By exploring four-fold and six-fold dilutions, we could span a wider range of concentrations on a single chip. For example, a two-fold dilution chip could span 75 to 300 µg/mL while a six-fold dilution chip could span 25 to 900 µg/mL (Supp. Fig. 3).

Prior to testing, dyes and antibitoics were mixed and immobilized on the chip by drying. No color change was seen in the center of the chip implying that the dye stayed well confined in the wells. As mentioned previously, the antibiotics may flow back into the center of the chip, but they do not impact adjacent wells. If antibiotics were influencing adjacent wells, the positive control would disappear, especially in the case of high susceptible samples, and the test would be invalidated. Therefore, since no mixing of antibiotics was observed, the antibiotic configurations can easily be changed based on clinical needs, such as local resistance trends or antibiotic availability.

Ciprofloxacin, meropenem, and gentamicin two-fold and four-fold dilutions had an average categorical agreement of 79% compared to 77% for six-step dilutions (Fig. 2b, Supp. Fig. 4). Cefazolin, tobramycin, and ampicillin had average accuracies of 68%, 78%, and 67% for two-fold, four-fold, and six-fold dilutions respectively (Fig. 2b). Therefore, we used four-fold dilutions in all future testing. Out of all the antibiotics, ciprofloxacin performed the best with over 92% accuracy in every dilution and dye tested. For context, the World Health Organization (WHO) is aiming for 90% accuracy with less than 5% major discrepancies for an ideal antibacterial susceptibility test^21^.

### XTT displays higher overall accuracy but with higher proportions of very major errors compared to PrestoBlue

We compared XTT and PrestoBlue side by side to identify any major differences in accuracies between the two dyes for each antibiotic. Meropenem performed significantly better when paired with XTT compared to PrestoBlue: Meropenem-Presto displayed only a 50% accuracy with 23% major discrepancies compared to 83% accuracy with no major discrepancies for Meropenem-XTT. We suspect this is due to a chemical interaction between meropenem and the resazurin component of PrestoBlue dye. Cefazolin, tobramycin, and ampicillin displayed significantly higher very major discrepancies rates using XTT dye. In all dilution cases, gentamicin displayed similar accuracies between PrestoBlue and XTT but had fewer very major discrepancies using PrestoBlue. Overall, XTT had better accuracy in general but at the expense of higher very major errors rates, which could lead to misprescribing of antibiotic therapies.

### Bac-PACs are shelf-life stable for 1 week at room temperature and 1 month under refrigeration

For clinical use, diagnostic assays are manufactured, transported, and stored for some amount of time prior to use. Therefore, the Bac-PAC was evaluated for storage temperature and shelf-life. The paper chips were prepared as in all previous experiments, dried at ambient air for approximately 10 mins, and placed in heat seal packaging with a single desiccator packet. Half of the chips were stored at room temperature (approximately 20 °C) while the other half were stored in the fridge (approximately 4 °C).

**Figure 3:**
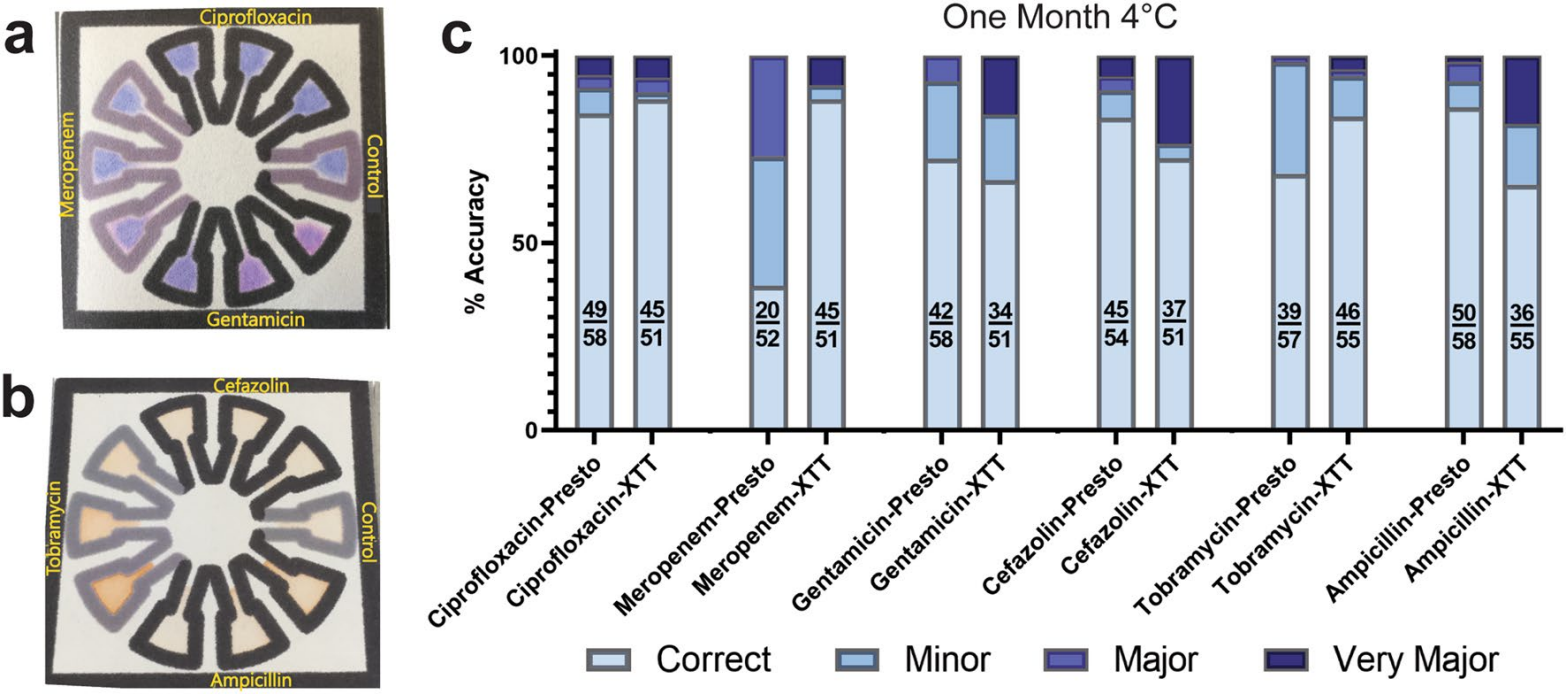
Shelf-life testing for 1 week and 1 month. (**a**) PrestoBlue with ciprofloxacin, meropenem, and gentamicin after 1 month at room temperature but before any bacterial loading. (**b**) XTT with cefazolin, tobramycin, and ampicillin after 1 month at room temperature but before any bacterial loading. (**c**) Ciprofloxacin, meropenem, gentamicin, cefazolin, tobramycin, and ampicillin results after 1 month of storage at 4 °C Fractions represent number of correct samples over number of total samples.

Chips stored at room temperature for one month displayed some color change even without bacteria. PrestoBlue appeared to be converted by the highest concentrations of meropenem and gentamicin (Fig. 3a) while XTT was readily converted by high levels of tobramycin and ampicillin (Fig. 3b). When bacterial samples were added, this effect increased the discrepancy rate (Supp. Figs. 5 and 6). This effect is unsurprising given that XTT and ampicillin are typically stored at − 20 °C while PrestoBlue, meropenem, gentamicin, cefazolin, and tobramycin are stored at 4 °C.

Chips stored in the fridge at 4 °C fared much better and retained higher accuracies after one month when compared to those at room temperature. In all cases except for meropenem, the choice of dye made little difference in the overall accuracy of the test. With meropenem, however, use of PrestoBlue lead to a very high minor and major discrepancy rate compared to XTT after one month of storage in the fridge (Fig. 3c). This is likely caused by high levels of meropenem converting PrestoBlue over time, resulting in false positives. This theory is further supported by the lack of very major discrepancies. The best antibiotic dye pairing results after one month of storage at 4 °C were Ciprofloxacin-XTT, Meropenem-XTT, and Gentamicin-Presto displaying 88%, 88%, and 72% accuracy, respectively, and Cefazolin-Presto, Tobramycin-XTT, and Ampicillin-Presto displaying 83%, 84%, and 86% accuracy, respectively. In the case of Meropenem-XTT, Ciprofloxacin-Presto, and Ciprofloxacin-XTT, storage at room temperature instead of refrigerated made little difference in the performance of the test (Supp. Fig. 6). Therefore, most antibiotic-dye combinations on Bac-PAC are stable for at least one month when stored at 4 °C, and some pairings could be stored at room temperature for shorter time periods, such as in resource-poor areas without reliable access to electricity.

### Rechargeable SmartMug can be used for low-cost incubation

**Figure 4:**
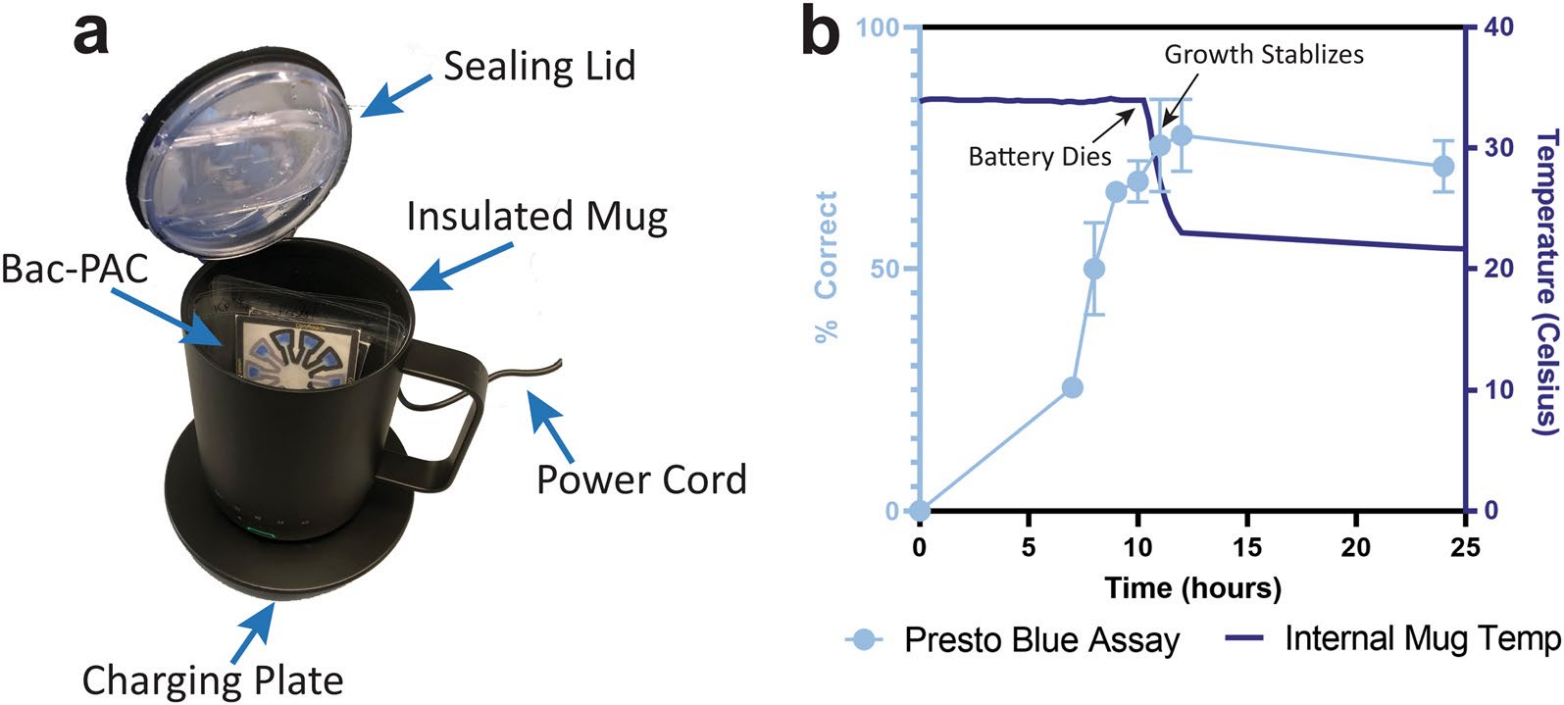
SmartMug incubator. (**a**) Picture of the Smart Mug rechargeable incubator system. (**b**) Accuracy of Bac-PACs in Smart Mug and temperature over 24 h when on battery power. Error bars show standard error of the mean.

While our results thus far are promising, they still rely on a large, water jacketed, energy intensive standing incubator which is not conducive to the point-of-care. Therefore, we substituted the 400 lb incubator for a 2.5 oz Smart Mug Warmer (Fig. 4a). The mug could maintain a constant 35 °C temperature and holding more than 30 Bac-PAC assays at a time. A significant change in assay accuracy was not seen when using the mug compared to the jacketed incubator as the heat source. Additionally, when fully charged, the mug could maintain a 34–35 °C temperature for 10–12 h, which was approximately the same time required for the PrestoBlue assays to reach their peak accuracies (Fig. 4b).

## Conclusions

The need for individualized AMR testing worldwide is expected to increase over the next decade. Traditional phenotypic culture methods for AMR, such as broth dilution and Kirby-Bauer disk diffusion, are costly, time consuming, and require trained personnel, making them inaccessible in low- and middle-income countries that will be hardest hit by drug resistant epidemics. Instead, the improved Bac-PAC system brings us closer to clinically actionable information at the point-of-care by being low-cost, disposable, easy to use, and portable.

While many studies have compared PrestoBlue and XTT in liquid culture^19^, relatively little is known about their applicability to paper diagnostics. While the XTT cell viability assay is commonly used in microbiology, it is typically suspended in liquid and read by spectrophotometry. PrestoBlue has been more commonly utilized for paper microfluidics^22^; however, our side-by-side comparison demonstrates that XTT may give more accurate antibiotic categorization under certain conditions. The use of six different antibiotics, all with accuracies of at least 75% with proper dye pairing, demonstrates the flexibility of this platform to be expanded to countless other antibiotics. This is on par with the E-test and agar dilution methods which display 90% and 79% agreeance respectively with broth microdilution^23^. It is also approaching the 90% accuracy level sought by WHO^21^. While many novel technologies are tested with only a handful species or strains, the utilization here of 30 bacterial strains representing 12 different bacterial species demonstrates the potential for this technology to be expanded to other urgent antibiotic resistant threats such as *Neisseria gonorrhoeae* and *Candida auris*^2^.

Other paper-based devices have been developed to diagnose bacterial infections^24–28^, detect viral infections^29^, and concentrate infectious DNA^30^, but only a few paper platforms perform some kind of susceptibility testing. Deiss et al.^31^ portable paper chip replicates the Kirby-Bauer disk diffusion method using PrestoBlue, but the readout requires measuring the zone of inhibition around each disk and comparing the results to published CLSI standards. Each chip also only provides the resistance profile for two antibiotics. Meanwhile, Michael et al.^32^ designed a fidget spinner like device capable of concentrating urinary pathogens and estimating their resistance using another visible dye, WST-8. While their AST readout takes approximately 2 h, it requires off-chip antibiotic exposure, multiple loading steps, separate devices for each antibiotic of interest, and has only been tested on *E. coli*. Meanwhile, the Bac-PAC is simpler to use with only one loading step and provides information on three antibiotics per device.

The Bac-PAC meets many of the priorities and goals for the development of accessible technologies for AMR outlined by the WHO^21^. For example, we calculated the disposable cost per chip as being $0.77 for XTT chips and $0.85 for the Presto Blue chips, well below the WHO recommended cost of $10–15 per assay^21^ (Supp. Fig. 8). Additionally, this assay can be used by an untrained user with a few hours of training, again below the WHO recommended user of a trained laboratory personal with 2 days of training. Not only is this test low-cost and easy to use, but also stable over one month when stored protected from light at 4 °C and for at least one week at 20 °C. Literature suggests that storage under nitrogen vacuum may extend this shelf life even further^33^. Finally, we drastically reduced capital costs by replacing the typical water jacketed incubator with a rechargeable, low power coffee mug that maintains the required 35 °C for at least 10 h on battery or indefinitely using a typical outlet. This approach meets the recommended < 25 kg weight, > 8-h battery life backup, and instrument costs of less than $10,000 also listed by WHO^21^. In the future, it may be feasible to replace the SmartMug incubator with a low-cost resistive microheater, further reducing cost and weight^34^.

Although this work is a promising proof of concept for a cheap, reliable, and accessible antibiotic susceptibility test, some limitations of the study should be noted. First, the panel of bacteria utilized in testing contained a higher portion of resistant samples than may be found in the general population. Additionally, pure bacterial samples were tested in spiked media. Further validation is need with multi-organism human samples. To eliminate these limitations, further studies should focus on clinical trials of real samples from a diverse patient population. Additionally, *Providencia stuartii* reads as susceptible to aminoglycosides in antibiotic susceptibility testing but is intrinsically resistant, and *Shigella sonnei* and *Salmonella Typhimurium* are not effectively treated with cephalosporins, such as cefazolin. Therefore, this test may need to be paired with a rapid bacterial identification strategy to eliminate these specific treatment options that are species dependent. In the future, we hope to incorporate direct from patient inoculation and rapid species identification to fully meet the WHO AMR criteria.

## Methods

### Paper microfluidic printing

A circular based paper microfluidic was designed in Adobe Illustrator 25.3.1 and printed on Whatman Grade 1 paper using a Xerox ColorQube 8570 wax printer. Wax was melted through the paper on a hot plate at 100 °C for ~ 30 s until the wax visibly melted through the paper. The back side of the chips was sealed with clear packing tape. Chips were stored in petri dishes at room temperature until use. The overall size of the device is 52 × 52 mm with 1 × 8 mm channels, 5 × 7 mm wells, and a 15 mm diameter central loading zone.

### Bacterial strains

Test strains were obtained from the Centers for Disease Control (CDC) and Food and Drug Administration (FDA) Antibiotic Resistance Isolate Bank^35^. We utilized 30 strains from the “*Enterobacterales* Carbapenem Breakpoint” panel containing 12 different species of Enterobacterales with varying levels of resistance to ciprofloxacin, meropenem, gentamicin, cefazolin, tobramycin, and ampicillin (Supp. Figs. 1–2, 9–11). Species included: *Escherichia coli*, *Enterobacter cloacae*, *Klebsiella pneumoniae*, *Klebsiella aerogenes, Citrobacter freundii, Citrobacter koseri, Providencia stuartii, Serratia marcescens, Klebsiella oxytoca, Proteus mirabilis, Shigella sonnei,* and *Salmonella Typhimurium.* Gold standard minimum inhibitory concentrations were obtained by the CDC/FDA Antibiotic Resistance Isolate Bank through broth microdilution^35^. Bacteria from freezer stocks were grown for 20–24 h on Mueller Hinton II agar and re-plated onto fresh BBL Mueller Hinton II agar and grown 20–24 h prior to testing. Bacterial strains were adjusting to a cell density of ~ 1 × 10^8^ using the optical density at 625 nm and diluted 1 to 10 in BBL Mueller Hinton II media prior to testing.

### Antibiotics

Gold standard antibiotic susceptible categories were assigned by the CDC/FDA Antibiotic Resistance Isolate Bank^35^ based on Clinical Laboratory Standard Institute’s M100 Performance Standards for Antimicrobial Susceptibility Testing^6^. Certified Pharmaceutical reference or secondary standards were used for all six antibiotics tested.

### Microfluidic device loading

Antibiotic stock solutions were diluted in 2 mg/mL XTT with 5 μg/mL phenazine methosulfate or pure PrestoBlue Cell Viability dye. The highest working concentration of antibiotic dye solution was diluted twice either 1 to 2, 1 to 4, or 1 to 6 depending on the trial requirements. 3μL of dye-only solution or antibiotic dilution were loaded on to the pre-printed and melted microfluidic chip starting at the 3 o’clock position and continuing from lowest to highest antibiotic concentration moving counterclockwise (Supp. Fig. 3). Dye and antibiotic solutions immobilized in the wells by drying at room temperature prior to testing. Multiplexed chips contained either ciprofloxacin, meropenem, and gentamicin or cefazolin, tobramycin, and ampicillin. 90 μL of diluted bacteria solution was placed in the center well of the chip and allowed to diffuse outward, filling all 10 outer wells. All experiments contained at least 2 replicates for each strain. 30 μL of sterile deionized water was placed in each corner of the chip to increase humidity. The chip was sealed between two half pieces of sterile ELISA sealing film by applying pressure with a finger around all the edges, labelled with sample name and antibiotics, and incubated overnight for at least 10 h at 37 °C (Supp. Video 1).

### Microfluidic device storage

For one-week and one-month storage trials, chips were prepared with the dye and antibiotic solutions as above. After fully drying at room temperature, chips were enclosed in an opaque heat-seal packaging with a single desiccator packet. Chips were then stored at either 4 °C or room temperature for the appropriate duration before use.

### Smart mug incubator

A VSITOO brand S3 Pro Smart Mug Warmer with Double Vacuum Insulation was purchased online. The mug was loaded with approximately 1 inch of water before adding Bac-PACs to prevent the mug automatic shutoff feature. Bac-PACs were prepared as usual and loaded vertically into the mug and the lid was sealed. Bac-PACs were removed at regular time intervals to check for growth and accuracy.

### Data analysis

Data was analyzed using GraphPad Prism 9 and Microsoft Excel 2016. For each antibiotic, the number of wells that did undergo a color change were recorded, where zero or one wells corresponded to a susceptible sample, two wells corresponded to an intermediate sample, and three wells corresponded to a resistant sample. Chips that did not undergo a color change in the dye-only positive control well were marked as incomplete. Percent accuracy was calculated as the number of correct susceptibility categorizations out of the total number of categorizations, once incomplete tests were removed. Percent “minor” discrepancies were calculated as the number of categorizations that were one category off (i.e., susceptible sample reading as intermediate) divided by the total number of categorizations, once incomplete tests were removed. Percent “major” discrepancies were calculated as the number of false-resistant results (i.e., susceptible sample reading as resistant) divided by the total number of categorizations, once incomplete tests were removed. Percent “very major” discrepancies were calculated as the number of false-susceptible results (i.e., resistant sample reading as susceptible) divided by the total number of categorizations, once incomplete tests were removed.

## Supplementary Information

Supplementary Figures.

Supplementary Video 1.

## Data Availability

All data supporting the findings in this study are available within the Article and its Supplementary Information.
