# Supplementary material for: Multiplexed paper-based assay for personalized antimicrobial susceptibility profiling of Carbapenem-resistant Enterobacterales performed in a rechargeable coffee mug
Source: Sci Rep. 2022 Jul 14;12:11990. doi: 10.1038/s41598-022-16275-3 (PMC9283407; doi:10.1038/s41598-022-16275-3)
Supplement: Supplementary file 1 — Supplementary Figures. [file 41598_2022_16275_MOESM1_ESM.docx]

**Supplementary Figure 1.** Bacterial Panel with gold standard MIC values from “Enterobacterales Carbapenem Breakpoint” from CDC/FDA Antibiotic Resistance Isolate Bank

**Supplementary Figure 2.** Bacterial species included in this study from the CDC/FDA AR Isolate Bank Panel “Enterobacterales Carbapenem Breakpoint”

**Supplementary Figure 3.** Concentrations used in 2-step, 4-step, and 6-step experiments Shown on the chips in the order they were applied and dried


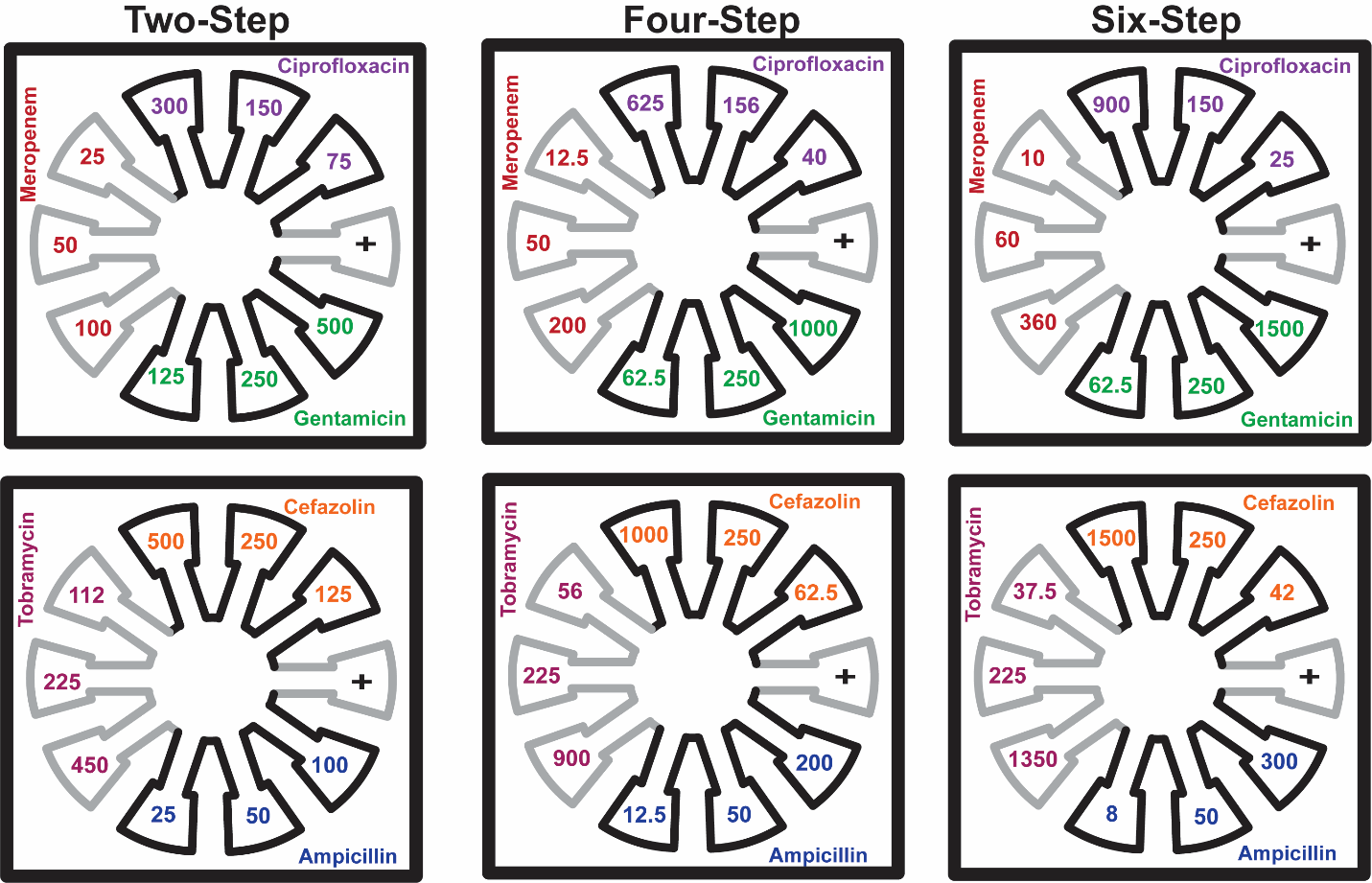


**
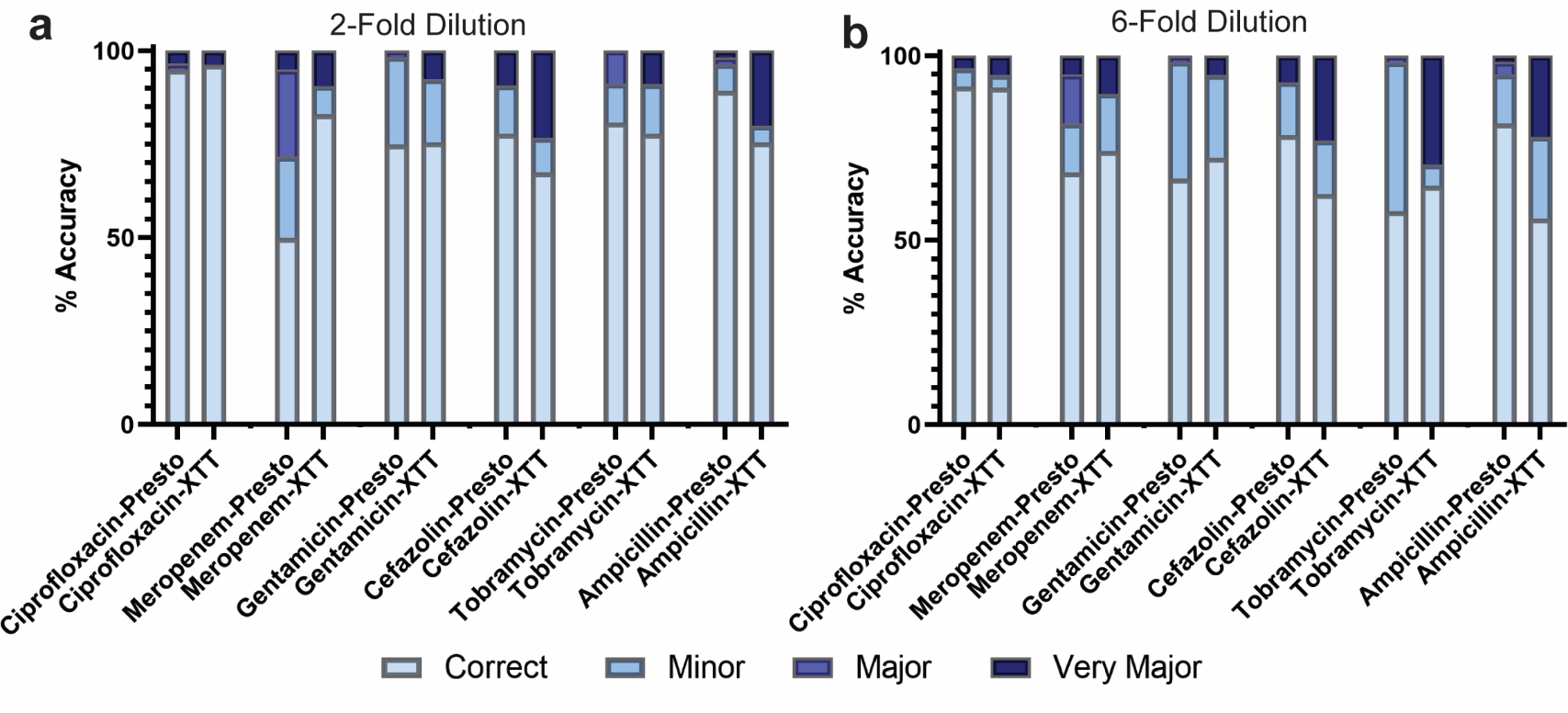
Supplementary Figure 4.** Results from diluting antibiotics a) 2-fold and b) 6-fold

**
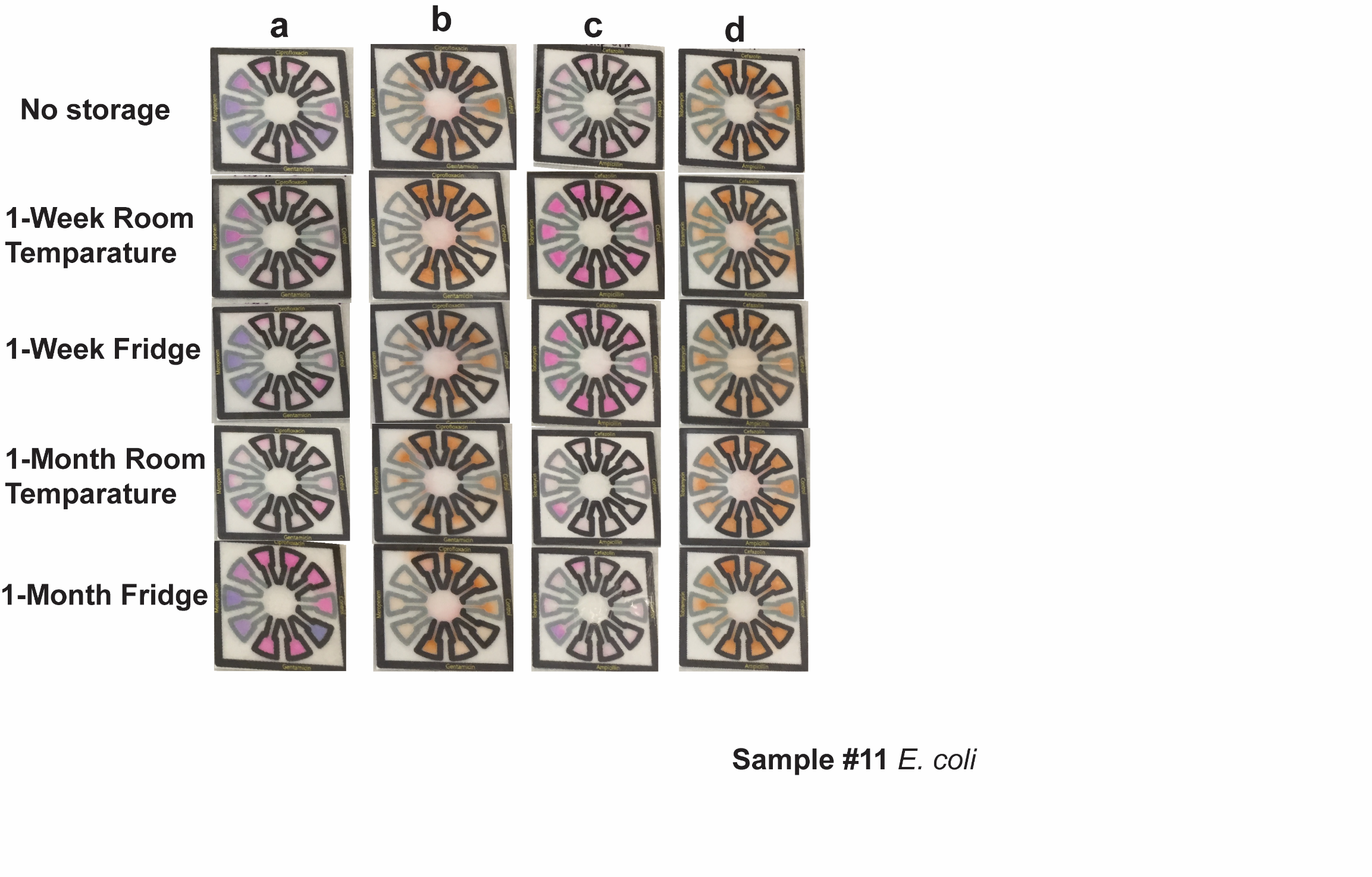
Supplementary Figure 5.** Example results of *E. coli* Sample AR#0011 with gold standard result susceptible to meropenem and resistant to ciprofloxacin, gentamicin, cefazolin, tobramycin, and ampicillin a) Ciprofloxacin, meropenem, and gentamicin with PrestoBlue b) Ciprofloxacin, meropenem, and gentamicin with XTT c) Cefazolin, tobramycin, and ampicillin with PrestoBlue d) Cefazolin, tobramycin, and ampicillin with PrestoBlue

**
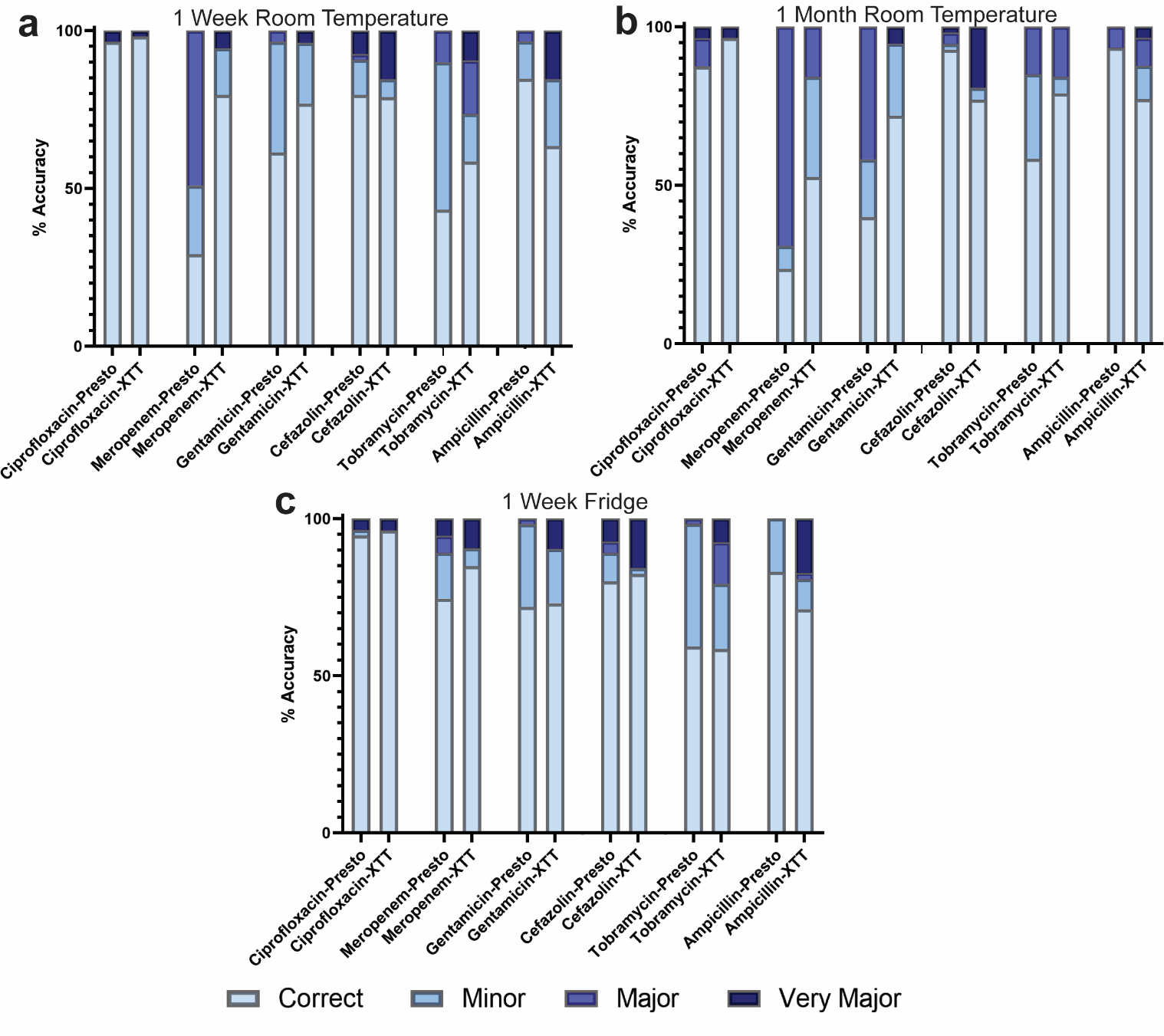
Supplementary Figure 6.** Additional Shelf-Life Storage Results a) 1 week stored at room temperature b) one month stored at room temperature c) one week stored in the fridge

**Supplementary Figure 7.** Spiked Human Urine Testing at 10^8^ - 10^5^ CFU/mL. a) 10^8^ CFU/mL in pure urine b) 10^7^ CFU/mL 10% urine 90% media c) 10^6^ CFU/mL 1% urine 99% media d) 10^5^ CFU/mL 0.1% urine 99.9% media


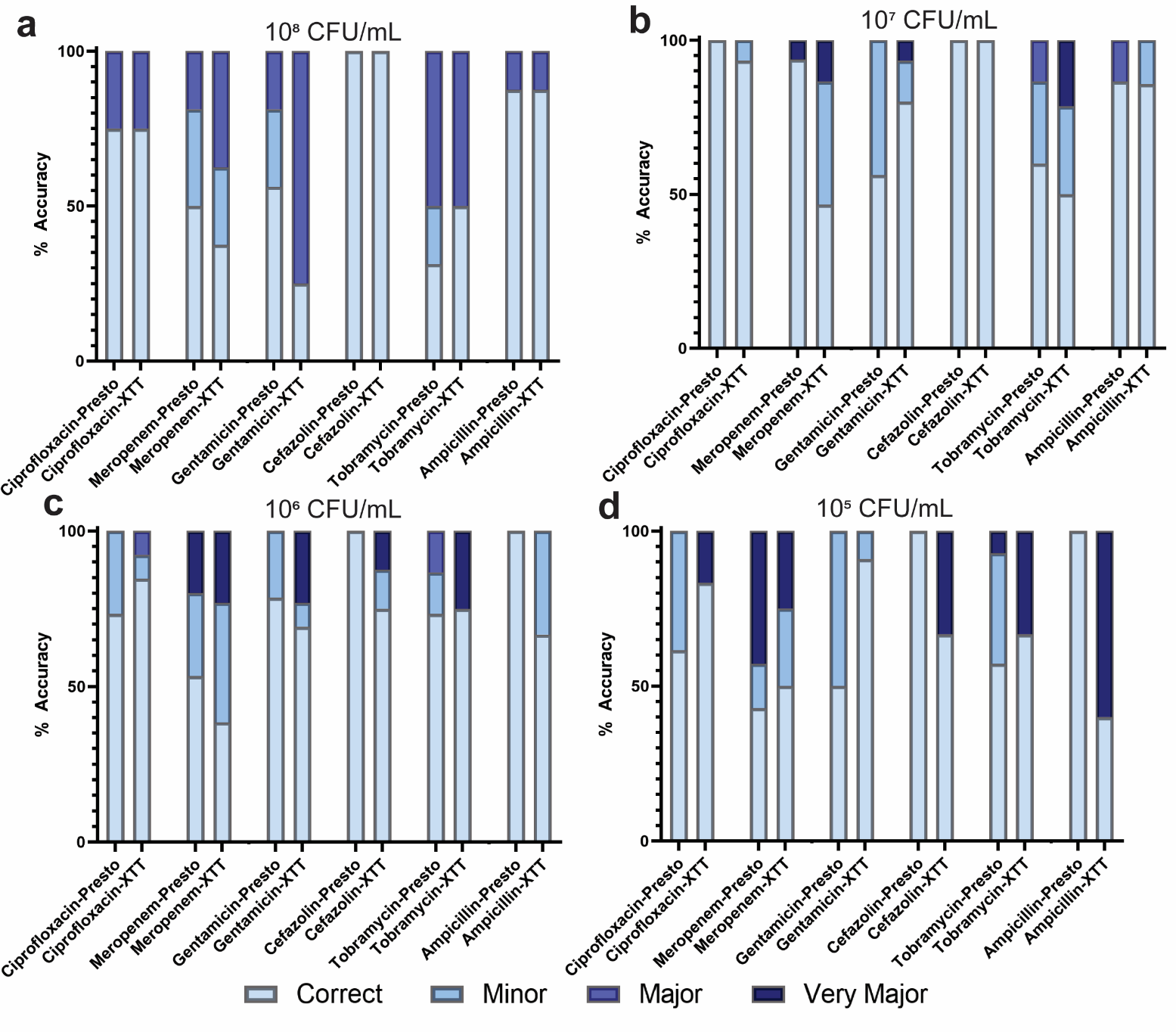


**Supplementary Figure 8.** Costs per Chip Including all disposables such as paper, sealing film, and pipette tips

| Item | Cost per unit | Unit/chip | Chip Cost |
| --- | --- | --- | --- |
| Grade 1 Chromatography Paper | $370/ 400 pages | 1/12 page | $0.08 |
| Wax Color Cubes | $72/100 pages | 1/12 page | $0.06 |
| PrestoBlue | $555/100mL | 0.030mL | $0.17 |
| XTT | $157/50mL | 0.030mL | $0.09 |
| PMS | $28/500mg | 0.05mg | < $0.01 |
| Ciprofloxacin | $74/1g | 820 µg/mL | < $0.01 |
| Meropenem | $203/500mg | 260 µg/mL | < $0.01 |
| Gentamicin | $76/1g | 1300 µg/mL | < $0.01 |
| Cefazolin | $163/500mg | 1300 µg/mL | < $0.01 |
| Tobramycin | $88/1g | 1200 µg/mL | < $0.01 |
| Ampicillin | $152/500mg | 260 µg/mL | < $0.01 |
| Mueller Hinton II Media | $62/500mL | 0.090mL | $0.01 |
| Packing tape | $6/40 yards | 0.05yards | $0.01 |
| Sealing Films | $43/100 | 1 | $0.43 |
| 100uL pipette tips | $40/960 tips | 2 | $0.08 |
| **Total** | **----** | **----** | **$0.77-$0.85** |

 **Supplementary Figure 9.** Data in table form for two-fold, four-fold, and six-fold dilutions

**Supplementary Figure 10.** Data in table form for 1-week and 1-month storage at room temperature and in fridge

**Supplementary Figure 11.** Data in table form for urine spiked samples
